# Development and validation of a preference based measure derived from the Cambridge Pulmonary Hypertension Outcome Review (CAMPHOR) for use in cost utility analyses

**DOI:** 10.1186/1477-7525-6-65

**Published:** 2008-08-21

**Authors:** Stephen P McKenna, Julie Ratcliffe, David M Meads, John E Brazier

**Affiliations:** 1 Galen Research Ltd, Enterprise House, Manchester Science Park, Lloyd Street North, Manchester, M15 6SE, UK; 2 School of Psychology, University of Central Lancashire, Preston, PR1 2HE, UK; 3 School of Health and Related Research, University of Sheffield, Regent Court, 30 Regent Street, Sheffield, S1 4DA, UK

## Abstract

**Background:**

Pulmonary Hypertension is a severe and incurable disease with poor prognosis. A suite of new disease-specific measures – the Cambridge Pulmonary Hypertension Outcome Review (CAMPHOR) – was recently developed for use in this condition. The purpose of this study was to develop and validate a preference based measure from the CAMPHOR that could be used in cost-utility analyses.

**Methods:**

Items were selected that covered major issues covered by the CAMPHOR QoL scale (activities, travelling, dependence and communication). These were used to create 36 health states that were valued by 249 people representative of the UK adult population, using the time trade-off (TTO) technique. Data from the TTO interviews were analysed using both aggregate and individual level modelling. Finally, the original CAMPHOR validation data were used to validate the new preference based model.

**Results:**

The predicted health state values ranged from 0.962 to 0.136. The mean level model selected for analyzing the data had good explanatory power (0.936), did not systematically over- or underestimate the observed mean health state values and showed no evidence of auto correlation in the prediction errors. The value of less than 1 reflects a background level of ill health in state 1111, as judged by the respondents. Scores derived from the new measure had excellent test-retest reliability (0.85) and construct validity. The CAMPHOR utility score appears better able to distinguish between WHO functional classes (II and III) than the EQ-5D and SF-6D.

**Conclusion:**

The tariff derived in this study can be used to classify an individual into a health state based on their responses to the CAMPHOR. The results of this study widen the evidence base for conducting economic evaluations of interventions designed to improve QoL for patients with PH.

## Background

Pulmonary hypertension (PH) is a disease characterized by a progressive rise in pulmonary artery pressure and pulmonary vascular resistance, ultimately resulting in right heart failure and death [1]. Symptoms include breathlessness, fatigue, palpitations, ankle oedema, chest pain, and syncope. Treatments for PH range from oral endothelin receptor antagonists through to nebulised or continuous intravenous or sub-cutaneous infusions of prostaglandin or prostaglandin analogues [2]. Many of these treatments are inconvenient or have significant adverse effects. For example, intravenous Prostacyclin [3] is associated with diarrhoea, systemic flushing, headaches, jaw pain and hypotension. Current treatments for PH (with the exception of pulmonary endarterectomy for thromboembolic PH) do not cure the disease.

The present aim of treatment is to lengthen survival time, to ameliorate symptoms and to improve quality of life (QoL). However, treatments for PH are expensive. For example, Epoprostenol costs up to £71,000 per patient per year in the UK [4]. Given this cost there is a need to determine the benefits of such treatment.

Several countries have produced guidelines for the conduct of economic evaluations in health care including Canada [5], Australia [6] and the UK [7]. All guidelines indicate that the preferred methodology is cost utility analysis (CUA) whereby the benefits of health care interventions are measured according to quality adjusted life years (QALYs). In addition, there is general agreement that where possible a generic preference based measure of health status based on general population values should be used to calculate QALYs. Generic preference based measures of health status include the EQ-5D [8], the SF-6D [9] and the HUI-3 [10]. However, for some specific clinical conditions generic measures may be considered inappropriate due to their lack of sensitivity and relevance [11,12]. Pulmonary hypertension represents such a condition. In addition, there is evidence that disease-specific utility measures are more responsive than generic ones [13,14].

Until recently only generic health status measures were available for assessing the impact of PH from the patients' perspective. The Cambridge Pulmonary Hypertension Outcome Review (CAMPHOR) was developed as a PH-specific measure to fill this gap [15]. It consists of three separate scales that are specific to PH; symptoms, functioning and QoL. The content of the measure was derived directly from PH patients and all scales have been shown to have good face and content validity, reliability, reproducibility and construct validity [15]. Furthermore, all scales have been shown to fit the Rasch model indicating that they represent unidimensional scales [15,16]. The analysis also shows how severe each item is in relation to the construct being measured. However, scores from such a measure cannot be used directly to undertake economic evaluations of treatments. First it is necessary to convert it into a preference based measure. The approach used in this paper has been developed in Sheffield and used in the construction of the SF-6D and King's Health Questionnaire [9,17].

An advantage of converting a disease-specific measure is that the resulting utility values calculated will be specific to the condition in question. If the source measure was carefully developed then all the items will be relevant to the respondents' condition and no important issue will have been omitted.

The purpose of the present paper is to describe the development and validation of a preference based measure from the CAMPHOR that would yield utility values for patients with PH and allow more accurate economic evaluations of PH treatments.

## Methods

### Item selection

As the ultimate purpose of the study was to calculate QALYs, it was decided to construct the preference based measure from the 25-item CAMPHOR QoL scale. Fewer items are included in a preference based measure as, otherwise, it would require an unmanageable number of valuations in order to determine the utility of all possible health states. Consequently, a simplified health state classification for CAMPHOR was developed based on a sample of six items. These six items were combined into four domains such that two domains had three levels and the other two domains had two levels. The items were selected by re-analysis of the responses of 201 patients to the CAMPHOR QoL scale. The following criteria were employed for item selection:

• Percentage affirmation of item: Items that were affirmed by a very small or very large proportion of the sample were excluded.

• Item severity as assessed by logit location in Rasch analysis: Items with extreme logit locations (derived from the Rasch analyses) were candidates for exclusion. However, it was the aim to include a reasonable spread of items in terms of the degree of severity they represented. The logit severity of the selected items ranged from -1.25 to 2.09.

• Regression: Ordinal regression was employed using CAMPHOR QoL responses to predict a general health perception variable (response options 'Very good', 'Good', 'Fair', and 'Poor'). Items that significantly predicted this variable were candidates for inclusion in the utility exercise.

• Content of item: In addition to the statistical methods listed above it was important to select items that covered a range of issues included in the CAMPHOR scale.

### Valuation survey

The main valuation survey was undertaken using the time trade-off (TTO) technique where individuals are asked to undertake conventional TTO valuations for a sample of health states. The Measurement and Valuation of Health (MVH) group version of TTO [18] was used to allow comparison with the EQ-5D tariff.

A representative sample of the adult general population was invited to participate in the study. Consenting adults were visited in their home for the TTO interview. A small pilot study (n = 15) was undertaken in advance of the main study to check that interviewees understood the task and were answering the questions as expected. The final sample size for this study was 249 individuals.

At the start of each interview respondents were given a self-completed questionnaire containing the EQ-5D and the CAMPHOR health state classification to complete. Respondents were then asked to rank the CAMPHOR health states from best to worst in order to help familiarize them with the states. The main elicitation task involved the use of a visual prop designed by the MVH group for use in the UK valuation of the EQ-5D. For health states that a respondent regards as better than being dead, they are asked to imagine two scenarios: 1) live in a state for 10 years (t) and 2) a shorter period (x) in perfect health. The time in the shorter state is varied until respondents are unable to choose between these two scenarios, at which point the value of the state is given as x/t. For states respondents regard as worse than being dead, the choice is between 1) dying immediately and 2) spending a period of time (x) in the state followed by (10-x) years in perfect health. Respondents were initially taken through a hypothetical TTO exercise to help them understand the task. They were then asked to undertake a total of nine TTO tasks. Finally, the interview concluded with a series of socio-demographic questions.

For states better than being dead, the value of the health state x/t is bounded by 1.0 for perfect health and zero for states as bad as being dead. For states worse than being dead, health state values were calculated using the formula -(10-x)/10 to ensure it is bounded by -1.0 [19].

### Modeling health state values

The data from the TTO interviews were analysed using two approaches based on aggregate and individual level modelling. First, ordinary least squares (OLS) were used to estimate a mean level model. The mean health state values were the dependent variable and the independent variables were a series of dummy explanatory variables representing each level of the CAMPHOR dimensions. The mean level model is defined as:

(1) *Yi *= *f*(*β*'**x**_*ij*_) + *Є*_*i*_

Where the dependent variable *Yi *is the mean TTO value for each health state and ***x ***is a vector of dummy explanatory variables (*x∂λ*) for each level *λ *of dimension *∂ *of the simplified CAMPHOR classification. For example, *x*_31 _denotes dimension *∂ *= 3 (dependence), level *λ *= 1 (I don't feel very dependent). For any given health state *x∂λ *will be defined as follows:

*x∂λ *= 1, if for this state dimension *∂ *is at level *λ*

*x∂λ *= 0, if for this state, dimension *∂ *is not at level *λ*

There are six of these terms in total with level *λ *= 1 acting as a baseline for each dimension. Hence for a simple linear model, the intercept (or constant) represents state 1111 and summing the coefficients of the 'on' dummies derives the value for all other states. *Є*_*i *_is the error term which is assumed to be independent with constant variance structure.

Secondly, a random effects model was used based on individual observations. This model specification takes account of the repeated measurement aspect of the data where multiple responses are obtained from the same individual.

The random effects model is defined as:

(2) *Yij *= *f*(*β*'***x***_*ij*_) + *Є*_*ij*_

Where i = 1,2...n represent individual health state values and j = 1,2...m represents respondents. The dependent variable *Yij *is the value assigned to each health state (i) valued by respondent j, ***x ***is a vector of dummy explanatory variables (*x∂λ*) defined as previously and *Є*_*ij *_is the error term which is subdivided as follows:

(3) *Є*_*ij *_= *u*_*j *_+ *e*_*ij*_

Where *uj *is respondent specific variation and *eij *is an error term for the *i*th health state valuation of the *j*th individual. This is assumed to be random across observations.

### Validation of the CAMPHOR preference based measure

After the valuation exercise it was possible to use the resulting weights for the six items to calculate utility data for previously collected CAMPHOR responses. Data collected in a previous study [15] were available to validate the new preference based measure (which is embedded in the CAMPHOR QoL scale). This study involved administering the CAMPHOR to 91 PH patients on two occasions, two weeks apart. In addition, the EQ-5D was administered on the second occasion. The following psychometric properties of the new measure were assessed; test-retest reliability (reproducibility) and construct validity (utility scores compared between perceived general health groups and between PH severity groups based on CAMPHOR symptom scores).

Ethical approval was sought and gained for the validation survey.

## Results

Table 1 includes details of items selected. The internal consistency for these six items was 0.72.

**Table 1:** Item selection details

**Item**	**%** ** affirmation**	**Corrected** **item-total** ** correlation**	**Factor** ** loading**	**Rasch** ** location**	**Mean** ** change**
7	32.2	.49	.54	0.84	0.07
12	65.9	.54	.60	-1.25	0.07
13	14.4	.30	.33	2.09	0.16
15	38.9	.64	.69	0.42	0.10
24	19.3	.46	.50	1.88	0.09
25	60.9	.64	.69	-1.00	0.00

### Derivation of health state classification

Four domains were captured using the six selected CAMPHOR items; social activities, travelling, dependence and communication. Two items each provided three levels for the social activities (I can join in activities with family and friends, I'm unable to join in activities with family and friends, I feel very isolated) and Travelling (Travelling distances is not a problem, Travelling distances is a problem, I am reluctant to leave the house) domains. One item each provided two levels for the Dependence (I don't feel very dependent and I feel very dependent) and Communication (I never find speaking too much of an effort and Sometimes it's too much effort to speak) domains. A full factorial design produced 36 health states for valuation. The health states were stratified into mild, moderate and severe classifications. A sample of health states defined by the CAMPHOR items can be seen in Table 2. The health states were chosen to reflect a range of possible health states defined by the classification rather than predominantly a 'good' or 'bad' selection of health states.

**Table 2:** Sample health states defined by CAMPHOR

I can join in activities with my family and friends
Travelling distances is not a problem
I feel very dependent
I never find speaking too much of an effort

I can join in activities with my family and friends
Travelling distances is not a problem
I feel very dependent
Sometimes it's too much effort to speak

I can join in activities with my family and friends
Travelling distances is a problem
I don't feel very dependent
I never find speaking too much of an effort

### Valuation survey

Descriptive characteristics of the respondents included in the valuation survey are included in Table 3. It can be seen that a majority of respondents were female, married and had experience of serious illness in their own families. Over 60% of respondents had education beyond the minimum school leaving age with over 40% holding a degree or equivalent professional qualification.

**Table 3:** Descriptive characteristics of respondents

**Characteristics**	**Respondents**	
	N	%
**Age**		
18–25	12	4.8
26 to 35	28	11.2
36 to 45	63	25.3
46 to 55	54	21.7
56 to 65	50	21.1
66+	42	16.9
**Gender**		
Male	96	38.6
Female	153	61.4
**Relationship status**		
Married/living with partner	192	77.1
Living alone	57	22.9
**Personal experience of serious illness**		
Yes	81	32.5
No	168	67.5
**Experience of serious illness in family**		
Yes	164	66.1
No	84	33.9
**Experience of serious illness in caring for others**		
Yes	119	48.0
No	129	52.0
**Main activity**		
Employed or self employed	140	56.2
Other	109	43.8
**Education after minimum school leaving age**		
Yes	151	60.6
No	98	39.4
**Degree or equivalent professional qualification**		
Yes	103	41.4
No	146	58.6

The health state values ranged from 0.770 to 0.156 and generally had fairly large standard deviations (ranging from 0.250 to 0.532).

Table 4 shows the results for the mean level and random effects main effects only models (models 1 and 2).

**Table 4:** Consistent Mean and Random effects model results

***Mean level – model 1***			***Random effects – model 2***	
Disvalue	Coef. (95% CI)	P value	Coef. (95% CI)	P value

Lev2 social act.	-0.297 (-0.345 to -0.249)	<0.001	-0.307 (-0.337 to -0.276)	<0.001
Lev3 social act.	-0.308 (-0.357 to -0.258)	<0.001	-0.304 (-0.334 to -0.274)	<0.001
Lev2 travelling	-0.202 (-0.250 to -0.154)	<0.001	-0.207 (-0.240 to -0.174)	<0.001
Lev3 travelling	-0.223 (-0.273 to -0.173)	<0.001	-0.205 (-0.235 to -0.175)	<0.001
Lev2 dependence	-0.147 (-0.188 to -0.108)	<0.001	-0.156 (-0.181 to -0.131)	<0.001
Lev2 speaking	-0.147 (-0.187 to -0.107)	<0.001	-0.141 (-0.166 to -0.116)	<0.001
Constant	0.962 (0.905 to 1.020)	<0.001	0.961 (0.907 to 1.015)	<0.001
*N*	34		249	
Adjusted R^2^	0.936		0.373	
Inconsistencies	0		2	
Mean absolute error	0.041		0.042	
No. > 0.05	12 (35%)		13(38%)	
No. > 0.10	2 (6%)		2 (6%)	
*Mean error*	0.000		0.004	
LB	8.582		10.711	

For the mean level model, all of the coefficients had the expected negative sign and were statistically significant (p < 0.01). The coefficient estimates also increased with absolute size as the level of each dimension worsened. The explanatory power of the mean level model was 0.936 which is very high indicating that the model is a good fit for the data. For the random effects model, the results were similar in that all the coefficients had the expected negative sign but differed from the mean level model as not all of the coefficients increased with absolute size as the level of each dimension worsened (namely the movement from level 2 to level 3 in social activities and the movement from level 2 to level 3 in travelling). In common with the mean level model, all of the coefficients were statistically significant (p < 0.01). The explanatory power of the random effects model (0.373) was, however, somewhat lower than that of the mean level model which was not surprising given the much larger number of actual data points which this model is aiming to fit. The predictive ability of the two models was quite similar with both models resulting in a similar proportion of errors greater than 0.05 (35% for the mean level model and 38% for the random effects models, respectively) and both models resulting in two predictive errors greater than 0.10.

In both mean and random effects models the predictions were unbiased (t-test) indicating that neither model systematically over or under estimated the observed mean value and the Ljung-Box (LB) statistics suggested that there was no evidence of auto-correlation in the prediction errors of both models, when the errors are ordered by actual mean health state valuation.

As the upper anchor for the analyses was perfect health and not the best state as defined by the CAMPHOR utility scale, the predicted utility value for the latter was less than 1. This was necessary since, for the purpose of calculating QALYs, results must lie on a scale where '1' is full health and '0' represents death. The predicted values of state 1111 is the constant term, which had values of 0.962 and 0.961 in the mean and RE models, respectively.

Table 5 presents examples comparing the predicted values according to each model and the actual values for each health state.

**Table 5:** Comparison of predicted and actual values for selected health state classifications: mean level (ML) and random effects (RE) models

**Health** ** State**	**Actual mean**	**Estimated mean** ** (ML) model**	**Estimated mean** ** (RE) model**
1111	N/A	0.962	0.961
1211	0.770	0.760	0.754
2121	0.515	0.517	0.498
2312	0.272	0.295	0.308
3322	0.156	0.136	0.155

### Validation of the preference based CAMPHOR scale

A majority (87.8%) of the 91 participants in the CAMPHOR validation survey were in New York Heart Association (NYHA) classes II and III. The correlation between the CAMPHOR QoL scores and the CAMPHOR preference based scores was 0.86.

### Test-retest reliability

After removal of cases where there were 7 days < or >21 days between administrations or where perceived health changed between administrations, the test-retest coefficient was 0.85. Tables 6 and 7 show how the preference weights are related to perceived general health and PH severity, respectively. In both cases Kruskal-Wallis tests showed that the differences in utility were statistically significant (p < .001).

**Table 6:** Association between preference weights and perceived general health

**Rating of general health**	**N**	**Utility**
Very good/good	35	0.69
Fair	36	0.49
Poor	16	0.37

**Table 7:** Association between preference weights and perceived severity of PH

**PH symptom severity**	**N**	**Utility**
Mild	18	0.90
Moderate	23	0.56
Quite severe	22	0.49
Very severe	23	0.31

Similar values were found for the mean preference weights obtained for the CAMPHOR and EQ-5D in NYHA Class II (Table 8). These values are also relatively similar to those found in a PH study that obtained utility values from the SF-6D [20]. However, there were marked differences between these three measures for Class III patients with the CAMPHOR utility scores being substantially lower than those on the EQ-5D and SF-6D. The CAMPHOR-generated preference weights showed greater sensitivity in terms of differentiating between NYHA classes. To illustrate this; if patients were to improve from NYHA Class III to Class II the effect size (difference in mean score divided by standard deviation at baseline) would be 0.71–0.92 for the CAMPHOR measure – a large effect size – compared with 0.42 for the EQ-5D. A moderately sized correlation (0.60) was found between the values derived from the two measures.

**Table 8:** Preference weights for NYHA Classes II and III

**Mean (SD) utility value**	**CAMPHOR**		**EQ-5D**	**SF-6D**
	Time 1	Time 2		

NYHA Class II	0.70 (0.24)	0.66 (0.29)	0.69 (0.24)	0.67 (0.10)
NYHA Class III	0.48 (0.24)	0.45 (0.24)	0.59 (0.24)	0.60 (0.10)
Difference	0.22	0.20	0.10	0.07
Effect size if patients move from Class III to Class II	0.92	0.71	0.42	0.70

## Discussion

The results from this study present a method for analysing existing and future data from clinical trials and other evidence sources where the CAMPHOR has been employed. Thus the CAMPHOR is now able to provide data on health state values in addition to PH-specific symptomatology, functioning and QoL. The methodology employed has produced preference data that can be applied within the framework of cost utility analysis in economic evaluation.

The mean level (model 1) is broadly consistent with a priori expectations in terms of coefficient size and direction of preference in relation to worsening levels of each dimension. The predicted health state values from this model also broadly conform to the logical ordering of the simplified CAMPHOR classification. This is not the case for the random effects model (model 2) which fails to indicate the direction of preference expected in terms of worsening levels of the social and travelling dimensions.

Hence, it is recommended that Model 1 (the aggregate mean level model) be used. This model is superior because it removes inconsistencies and because of its high performance in terms of explanatory power and predictive ability. The tariff can be applied by classifying individuals into particular health states on the basis of their responses to the CAMPHOR. For example, an individual who indicates that they can join in activities with family and friends, that travelling distances is a problem, that they feel very dependent, and sometimes find it too much of an effort to speak, would be classified in health state 1222. The corresponding value for that health state according to the recommended model is 0.465. All other health state classifications arising from the CAMPHOR can be valued using the same approach.

The CAMPHOR preference-based measure exhibited high correlations with the CAMPHOR QoL scale. High test-retest values indicate that the new utility scale has excellent reproducibility while evidence of the scale's validity was found in its ability to discriminate effectively between patients who have differing levels of disease severity.

The estimation of preference weights for disease-specific QoL instruments is relatively rare and some health economists have expressed scepticism about the value of such an exercise [21]. However, the main argument for using disease-specific descriptive systems rests on the premise that they are far more likely to be sensitive to changes in the condition under consideration (supported by results from the present validation exercise) and are more relevant to the concerns of patients than generic measures [22,23]. The effect size results show that the disease-specific measure is better able to distinguish signal from noise than the generic measures. This has important implications for sample sizes in trials. While it is accepted that for use in economic evaluation it is the absolute difference and not the effect size that determines cost effectiveness, the standard deviation influences the degree of uncertainty in the probabilistic sensitivity analysis [24].

There may also be a concern that the values produced by a disease-specific measure will not be comparable to those produced by a generic measure. However, it can be contended that providing the descriptive system is valued on the same scale using the same variant of the same valuation technique, as was the case for the CAMPHOR and EQ-5D models, then the valuations should be comparable [25].

The valuation exercise found that the best state defined by the CAMPHOR items was below 1. It is clear from this and other studies that individuals valuing the best health state (i.e. that with no health problems) are still judged to have impaired health status as reflected by a mean utility value lower than 1 [26,27]. It is interesting to note that in the development of the EQ-5D the state of perfect health was not valued and was assumed to be 1 [19]. This anchoring meant that in the PH validation sample around 8% of patients had perfect health according to the EQ-5D. It is questionable whether any individuals with PH would consider themselves to have perfect health given the severe nature of the symptoms, the fact that the condition is often not diagnosed until late in its progression and the poor prognosis. Given these factors, it is possible that the CAMPHOR utility scale provides a more realistic estimate of utility in PH.

## Conclusion

This research has demonstrated that it is possible to estimate preference weights for a disease-specific measure relating to pulmonary hypertension. The results can be applied to any data set including the CAMPHOR and hence widen the evidence base for conducting economic evaluations of new pharmaceuticals and other health care interventions designed to improve QoL for patients living with this serious condition.

The CAMPHOR preference-based measure has been shown to have very good psychometric properties. It has excellent reproducibility, good construct validity and superior sensitivity to the EQ-5D in this population.

## Competing interests

The study was sponsored by Actelion pharmaceuticals. Actelion may use the utility values derived in the study for cost-utility analyses relating to pulmonary hypertension treatments that they produce. Stephen McKenna and David Meads work for Galen Research Ltd who have, in the past, received other research funding from Actelion. A license is required for the commercial use of the CAMPHOR.

## Authors' contributions

SM designed and managed the study, identified the items for the valuation exercise and wrote the manuscript. JR designed and managed the valuation survey, conducted analysis and reported on the valuation exercise. *DM ran the analysis to identify items for the valuation exercise, analysed the validation data and contributed to the writing of the manuscript. JB designed and managed the valuation survey, analysed and reported on the valuation data and contributed to the writing of the manuscript. All authors read and approved the final manuscript.
